# *In Vivo* Messenger RNA Introduction into the Central Nervous System Using Polyplex Nanomicelle

**DOI:** 10.1371/journal.pone.0056220

**Published:** 2013-02-13

**Authors:** Satoshi Uchida, Keiji Itaka, Hirokuni Uchida, Kentaro Hayakawa, Toru Ogata, Takehiko Ishii, Shigeto Fukushima, Kensuke Osada, Kazunori Kataoka

**Affiliations:** 1 Division of Clinical Biotechnology, Center for Disease Biology and Integrative Medicine, Graduate School of Medicine, The University of Tokyo, Bunkyo-ku, Tokyo, Japan; 2 Department of Bioengineering, Graduate School of Engineering, The University of Tokyo, Bunkyo-ku, Tokyo, Japan; 3 Department of Rehabilitation for the Movement Functions, Research Institute, National Rehabilitation Center for the Persons With Disabilities, Tokorozawa, Saitama, Japan; 4 Department of Materials Engineering, Graduate School of Engineering, The University of Tokyo, Bunkyo-ku, Tokyo, Japan; Consejo Superior de Investigaciones Cientificas, Spain

## Abstract

Messenger RNA (mRNA) introduction is a promising approach to produce therapeutic proteins and peptides without any risk of insertion mutagenesis into the host genome. However, it is difficult to introduce mRNA *in vivo* mainly because of the instability of mRNA under physiological conditions and its strong immunogenicity through the recognition by Toll-like receptors (TLRs). We used a novel carrier based on self-assembly of a polyethylene glycol (PEG)-polyamino acid block copolymer, polyplex nanomicelle, to administer mRNA into the central nervous system (CNS). The nanomicelle with 50 nm in diameter has a core-shell structure with mRNA-containing inner core surrounded by PEG layer, providing the high stability and stealth property to the nanomicelle. The functional polyamino acids possessing the capacity of pH-responsive membrane destabilization allows smooth endosomal escape of the nanomicelle into the cytoplasm. After introduction into CNS, the nanomicelle successfully provided the sustained protein expression in the cerebrospinal fluid for almost a week. Immune responses after mRNA administration into CNS were effectively suppressed by the use of the nanomicelle compared with naked mRNA introduction. *In vitro* analyses using specific TLR-expressing HEK293 cells confirmed that the nanomicelle inclusion prevented mRNA from the recognition by TLRs. Thus, the polyplex nanomicelle is a promising system that simultaneously resolved the two major problems of *in vivo* mRNA introduction, the instability and immunogenicity, opening the door to various new therapeutic strategies using mRNA.

## Introduction

Messenger RNA (mRNA) has a high potential to produce proteins or peptides for therapeutic purposes in a safe manner without any risk of random integration into the genome. Although pioneering studies to transfect mRNA into cells using a nonviral method were reported in the 1980s [1], [2], the interest in the clinical use of mRNA has been limited for a long time. There are two major problems associated with mRNA introduction: mRNA is considered to be unstable to obtain sufficient protein expression in clinical settings [3] and mRNA induces strong immune reactions through recognition by Toll-like receptors (TLRs) [4], [5], hampering repeated mRNA administration. Thus, efforts for clinical applications of mRNA have been limited, mainly in cancer immunotherapy by *ex vivo* transfection toward dendritic cells [6], [7], [8], [9]. In contrast, there are only a few studies reporting the trials of *in vivo* mRNA administration [10], [11].

Instability is an inherent limitation of mRNA. Many *in vitro* transfection studies have revealed that although mRNA enabled even higher efficiency of protein expression than plasmid DNA (pDNA) within several hours after mRNA introduction into cells, the duration of expression was apt to be very short [12], [13]. For example, several groups recently induced pluripotent stem cells (iPSCs) by transfection of mRNA encoding Yamanaka factors [14], [15], [16]. Their success strongly suggests the feasibility of using mRNA for therapeutic purposes in the future; however, they generally performed repeat transfections with intervals of a few days, suggesting that the instability of mRNA hampered the durable protein expression after mRNA transfection.

Thus, the requirement of an effective mRNA delivery system to overcome the instability of mRNA should be further explored to realise *in vivo* mRNA administration. Although some strategies have been reported for nonviral *in vivo* mRNA administration, including injection of naked mRNA [17], [18] in combination with physical pressure such as electroporation or gene gun [1], [19] and the usage of synthetic carriers based on cationic lipids and polymers [20], [21], [22], low efficiency and short duration of protein expression remain significant problems to be solved. At present, there is only one phase 1 study clinical trial to treat metastatic melanoma by subcutaneous injection of naked or protamine-stabilised mRNAs [9]; however, a more efficient system for *in vivo* mRNA administration would be strongly required to expand the application to many other clinical fields.

In addition to the stability issue, the problem of mRNA immunogenicity also remains unsolved. Based on findings that mRNA containing modified nucleosides effectively suppresses recognition by TLRs [23], [24], mRNA modification was proposed as an effective method to reduce immunogenicity. Several protocols for mRNA modification have been reported to effectively regulate the induction of inflammatory cytokines after mRNA administration, for example, replacement of uridine with pseudouridine [11], [25] or replacement of 25% uridine and cytidine with 2-thiouridine and 5-methyl-cytidine [10]. However, cytokine induction was not completely eliminated even when using modified mRNA. Moreover, the modified forms of pseudouridine and thiouridine are rarely found in endogenous mRNA [26], leaving their clinical safety and availability unclear.

These issues motivated us to apply a new methodology using our original nonviral carrier, polyplex nanomicelle [27], for *in vivo* mRNA administration. As a result of its characteristic core–shell architecture based on the self assembly of block copolymers composed of polyethylene glycol (PEG) and polyamino acids, the polyplex nanomicelle has a strong potential to function as an effective mRNA-containing carrier with high stability and stealth properties, thereby simultaneously addressing the issues of instability and immunogenicity of mRNA.

To evaluate polyplex nanomicelle capacity, the central nervous system (CNS) was targeted. For treatments of chronic neurogenic disorders and spinal cord injuries, continuous administration of therapeutic proteins and peptides into the intrathecal space has many potential applications. The proteins and peptides are able to move along perivascular spaces and axon tracts into the spinal cord, avoiding the blood–brain barrier (BBB), the major obstacle to therapeutic delivery into CNS [28]. However, continuous delivery of proteins and peptides generally requires physical means such as indwelling catheters, which often involve many risks and complications. Secreted transgene products from either pDNA or mRNA that are introduced into the neural tissues are promising alternatives. In particular, mRNA is a strong candidate because there is no risk of random integration.

In this study, we applied the polyplex nanomicelle system using a polycation, poly[N′-[N-(2-aminoethyl)-2-aminoethyl]aspartamide] ([PAsp(DET)]), for mRNA administration into CNS [29], [30], [31]. The system was discovered to have a high capacity for enhanced endosomal escape because of pH-responsive membrane destabilization by [PAsp(DET)] [32] as well as the unique character of rapidly degrading into nontoxic forms under physiological conditions, thereby minimizing cell damage and toxicity that incidentally occur after introduction in a time-dependent manner [33], [34]. By intrathecal injection into CNS, the feasibility of using the nanomicelle for *in vivo* mRNA administration was investigated through comprehensive analyses of continuous protein expression and regulated immunogenicity.

## Results

### Polyplex nanomicelle allowed *in vivo* mRNA introduction into CNS

**Figure 1:**
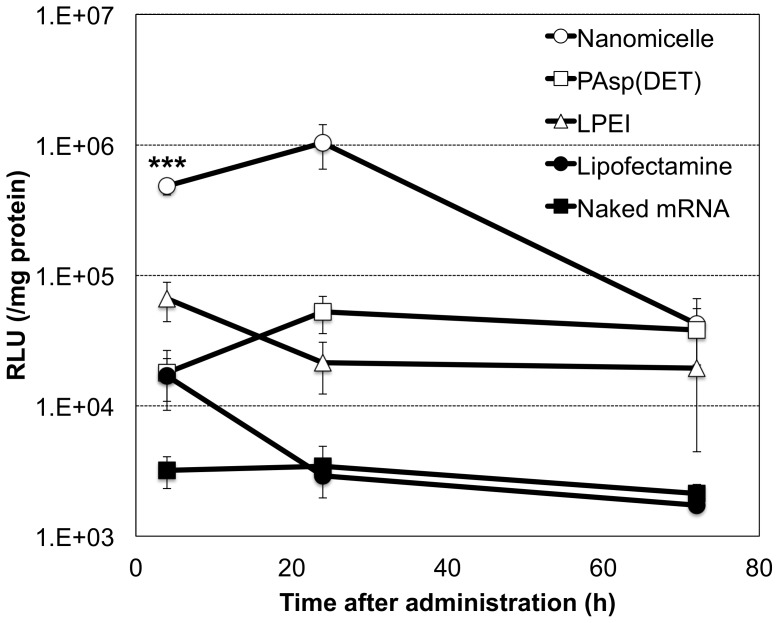
Time dependent profile of luciferase expression in CNS after mRNA administration. Ten µl of mRNA-containing carriers (2 µg mRNA) of polyplex nanomicelle composed of PEG–PAsp(DET) (open circle), polyplex formed with cationic polymer, PAsp(DET) (open square) and linear polyethyleneimine (LPEI) (open triangle), Lipofectamine 2000 (closed circle) and naked mRNA solution (closed square) were injected into the cisterna magna of mice. Luciferase expression was evaluated from the extracted brain and spinal tissues. The data are presented as the mean ± standard error of the mean (s.e.m.) (N≥5). Statistical significance was assessed by 2-tailed Student's t-test, ***, P<0.001 versus PAsp(DET), LPEI, Lipofectamine 2000 and naked mRNA groups. RLU, relative luminescence units.

**Figure 2:**
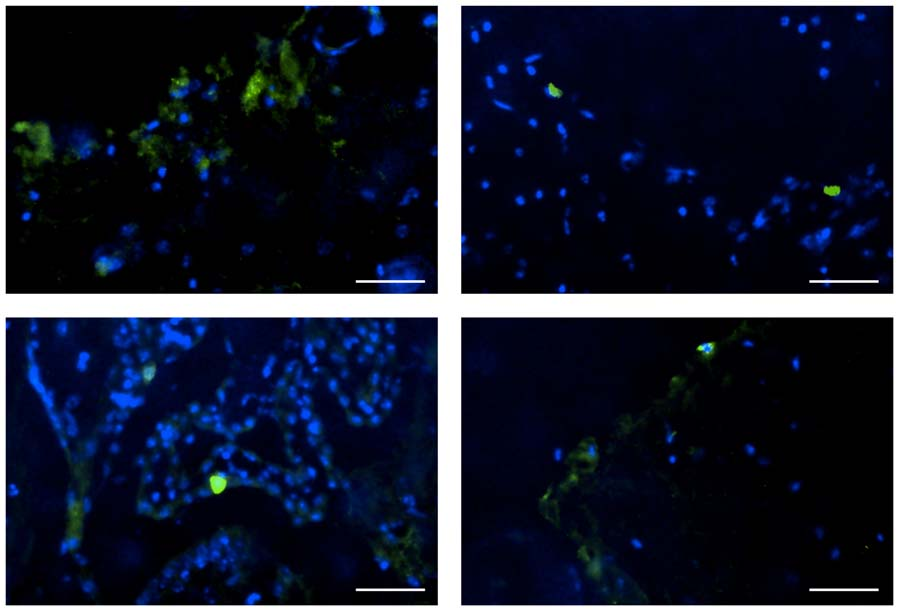
GFP expression in CNS after mRNA delivery using polyplex nanomicelle. Fluorescent microscopic images of brain tissue were taken 48 h after the administration of nanomicelle loading GFP-expressing mRNA. GFP was visualised by immunostaining using an anti-GFP monoclonal antibody (green). The cell nuclei were stained with Hoechst 33342 (blue). Scale bars: 20 µm.

First, luciferase-expressing mRNA with nucleoside modification was introduced into CNS using various carriers by intrathecal injection into the cisterna magna of mice. Luciferase expression was evaluated from extracts of the brain stem and surrounding neural tissue. Among the carriers, the polyplex nanomicelle with 50 nm in diameter (Fig. S1) composed of PEG–PAsp(DET) showed significantly higher luciferase expression than any other carriers including lipoplex (Lipofectamine 2000) and polyplexes without PEG shielding (Fig. 1). Luciferase expression by the nanomicelle was detected as early as 4 h after introduction and lasted for more than 24 h. Concomitantly, immunohistological analysis of CNS after introducing green fluorescent protein (GFP)-expressing mRNA using the polyplex nanomicelle revealed strong protein expression in meninges flanking the subarachnoid space (Fig. 2). We also analysed luciferase expression in other CNS sites away from the injection site of the occipital region. The expression was clearly detected from the brain to the lumbar region, strongly suggesting that the nanomicelle was widely distributed through the subarachnoid space (Fig. S2).

### Polyplex nanomicelle effectively regulated the immunogenicity of mRNA

**Figure 3:**
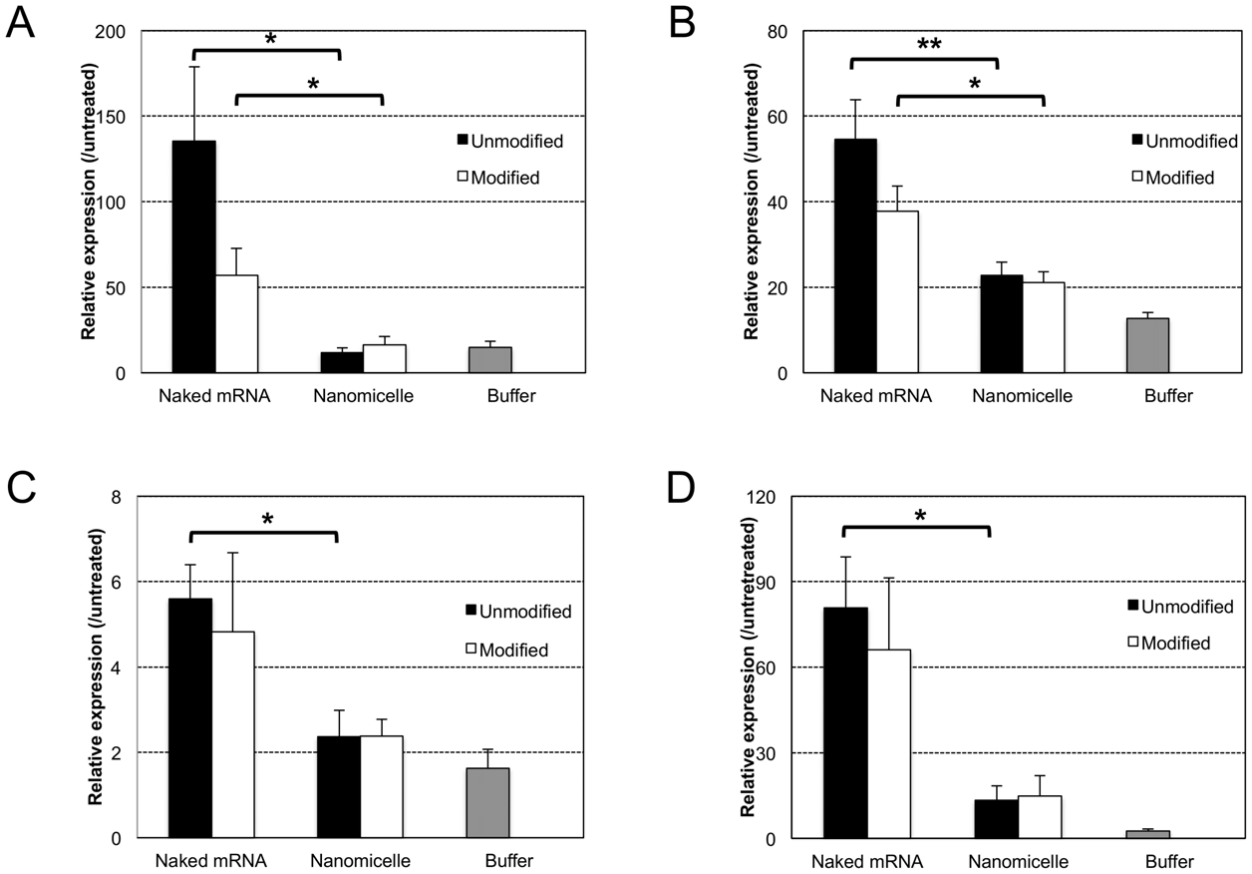
Evaluation of immune responses in CNS after mRNA delivery. mRNA (modified or unmodified) was administered into CNS either as a form of naked mRNA or using polyplex nanomicelle. Expression of proinflammatory cytokines of (a) interleukin (IL)-6, (b) tumour necrosis factor (TNF)-α, (c) interferon (lFN)-α4 and (d) IFN-β1 in the brain stem were measured using real-time quantitative PCR (RT-PCR) 4 h after administration. The data are presented as the mean ± standard errors of the mean (s.e.m.) (N≥4). Statistical significance was assessed by 2-tailed Student's t-test, *, P<0.05, **, P<0.01.

As mentioned in the Introduction, the immunogenicity of mRNA is a critical issue to achieve its effective and practical delivery into the body. This issue was addressed by analysing immune responses, including the induction of proinflammatory cytokines and type 1 interferon in CNS, after intrathecal injection of mRNA (modified or unmodified) in the form of naked mRNA or using the polyplex nanomicelle. Measurement of cytokines and type 1 interferons by quantitative polymerase chain reaction (qPCR) of total mRNA extracted from the brain stem and surrounding neural tissue clearly demonstrated that the immune responses after mRNA introduction were remarkably reduced by the use of polyplex nanomicelle compared with the administration of naked mRNA (Fig. 3). Indeed, naked mRNA induced significant immune responses, although it provided almost no luciferase expression in CNS (Fig. 1). Note that cytokine production can properly be evaluated from the expression level of corresponding mRNAs by qPCR method according to the literature [35], [36].

Comparison of modified and unmodified mRNA possessing identical sequences revealed that immune responses induced by naked unmodified mRNA were higher than those produced by modified mRNA (Fig. 3), confirming the ability of modification to reduce the immunogenicity of mRNA. However, the polyplex nanomicelle effectively suppressed immune responses even when using unmodified mRNA to the same extent as the modified mRNA.

For detailed evaluation of intracellular mechanisms, we focused on immune responses induced directly by recognition of mRNA by the innate immune systems. Exogenous mRNA is known to be recognised by TLRs, in particular, by TLR3, 7 and 8, that localize mainly on the membrane of the endosomes [37]. For this analysis, transformants of HEK293 cells that stably express a specific type of human TLR were used for *in vitro* mRNA transfection [23]. Since wild-type HEK293 cells have very low expression levels of endogenous TLRs, the transformants allowed us to analyse the specific recognition between exogenous mRNA and the specific type of TLR to induce the immune responses.

**Figure 4:**
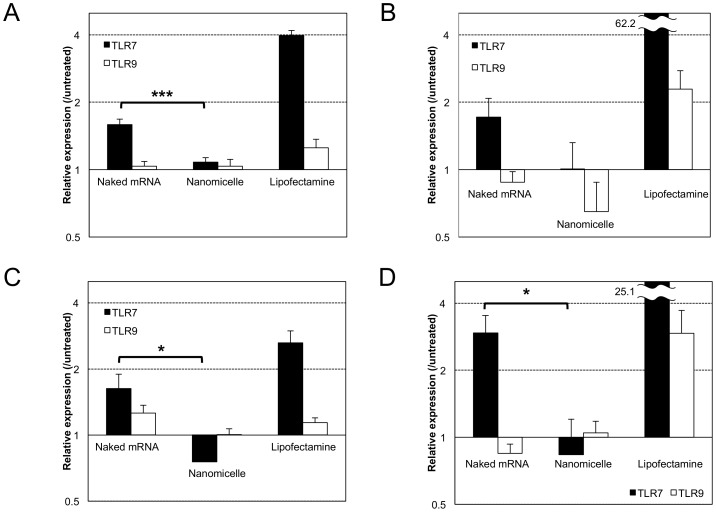
In vitro analysis of Toll-like receptor (TLR) signalling after mRNA introduction. To evaluate mRNA-mediated TLR signaling, HEK293 cells expressing TLR7 (grey bars) were used. HEK293 cells expressing TLR9 (white bars), which does not recognise mRNA, were also used as a negative control. Cells were treated with naked mRNA, polyplex nanomicelle or Lipofectamine 2000 using unmodified mRNA (a, b) or modified mRNA (c, d). Expression of interleukin (IL)-8 (a, c) and interferon (IFN)-β1 (b, d) was measured at transcriptional levels using real-time quantitative PCR (RT-PCR) 4 h after mRNA introduction. The data are presented as the mean ± standard error of the mean (s.e.m.) (N = 6). Statistical significance was assessed by 2-tailed Student's t-test, *, P<0.05, ***, P<0.001.

After transfection of mRNA (modified or unmodified) toward HEK293 transformant expressing human TLR7 (293-hTLR7), expression levels of inflammatory cytokine (IL-8) and type 1 interferon (IFN-β1) were measured 4 h after transfection. When using the nanomicelle, expression of IL-8 and IFN-β1 remained to the levels of nontransfected controls (Fig. 4). In contrast, after transfection of mRNA using Lipofectamine 2000 or as naked mRNA, significantly higher expression of IL-8 and IFN-β1 was induced in 293-hTLR7 cells. To confirm the specificity of mRNA recognition by TLR7, another type of transformant expressing human TLR9 (293-hTLR9), known to have specific affinity to double-stranded DNA (dsDNA) but not to mRNA [38], was used. For the 293-hTLR9 cells, almost no increase in expression of IL-8 and IFN-β1 was observed after transfection using naked mRNA as well as the nanomicelle (Fig. 4). In contrast, when using Lipofectamine 2000, IL-8 and IFN-β1 expression showed considerable upregulation compared with the controls, although the levels were remarkably lower than those in 293-hTLR7 cells. These tendencies were similarly observed for both modified and unmodified mRNA regardless of the transfection methods used.

To exclude the possibility that the differences in immune responses among the transfection methods were because of the differences in the amounts of cellular uptake of exogenous mRNA, we quantified the amounts by real-time quantitative PCR (RT-PCR) of total mRNA extracted from the transfected cells. Indeed, the amount of mRNA detected 4 h after transfection using the nanomicelle and Lipofectamine 2000 were approximately 1%–10% of the total dose of transfected mRNA, whereas the amount introduced by naked mRNA transfection was much lower by 4 digits (Fig. S3). Thus, these results strongly suggest that mRNA introduction in the form of naked mRNA as well as the introduction using Lipofectamine 2000 induced immune responses by recognition of mRNA by TLRs, where the degree of immune responses was a reflection of the amount of mRNA internalization into the cells. In contrast, the nanomicelle significantly reduced the mRNA-specific activation of TLR7 signalling even though the substantial amount of mRNA was internalised into the cells, indicating that the recognition of mRNA by TLR7 was effectively avoided by the use of nanomicelle.

### Prolonged protein secretion into cerebrospinal fluid (CSF) was achieved by mRNA introduced by polyplex nanomicelle

Finally, we evaluated the properties of mRNA for prolonged protein secretion into CSF. mRNA expressing Gaussia luciferase (GLuc), a secreted type of luciferase, was incorporated in the polyplex nanomicelle and introduced into the subarachnoid space of rats by intrathecal injection. GLuc expression in the CSF was then measured [39]. For comparison, pDNA expressing GLuc was also examined after incorporation in the polyplex nanomicelle. In addition, the proteinous form of GLuc was used after being collected from GLuc-expressing culture cells (Methods).

**Figure 5:**
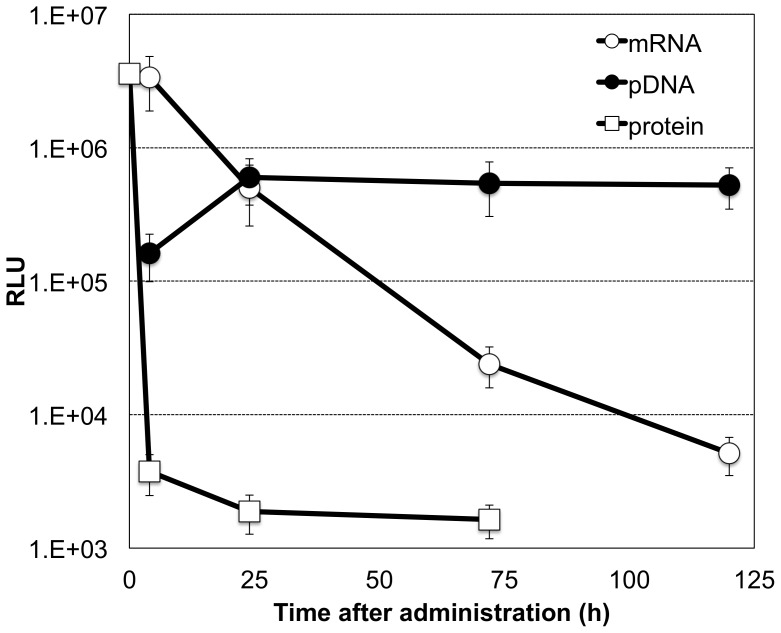
Comparison of mRNA, plasmid DNA (pDNA) and protein. mRNA or pDNA that expressed GLuc was incorporated in the polyplex nanomicelle and injected into the subarachnoid space of rats by intrathecal injection. The proteinous form of GLuc was also used for intrathecal injection. GLuc expression was evaluated from cerebrospinal fluid (CSF) collected at the indicated time points. The data are presented as the mean ± standard error of the mean (s.e.m.) (N = 4). RLU, relative luminescence units.

Time-dependent profiles of GLuc expression in CSF revealed that mRNA incorporated in the nanomicelle provided detectable expression in a sustained manner up to 120 h after introduction (Fig. 5). In contrast, after introduction of GLuc protein into the subarachnoid space, the protein rapidly decreased below detectable levels within 4 h after the introduction. pDNA showed prolonged GLuc expression for more than 120 h; however, mRNA showed 1-order higher GLuc expression 4 h after introduction.

## Discussion

This study represents the advantages of the polyplex nanomicelle containing mRNA for providing therapeutic proteins and peptides to CNS by intrathecal injection. Various recombinant proteins are available and in use for clinical purposes; however, the functional duration of the proteins is very short because of their poor stability under physiological conditions, leading to inconsistent outcomes. Moreover, repeated protein administration is costly. Compared with *in vivo* protein delivery, mRNA is apparently advantageous for obtaining protein secretion in a sustained manner with much less frequent administration.

It is reasonable to claim that pDNA has an advantage over mRNA from the viewpoint of sustainable transgene expression because DNA is much more stable than mRNA under physiological conditions. However, pDNA introduction is destined to cause an inevitable risk of random integration into the genome because the risk cannot be decreased below the level of spontaneous genetic recombination. DNA introduction may be accepted only for cases such as fatal diseases in which the risk of random integration is compensated by the benefit of DNA introduction, leading to difficulty in clinical applications of DNA introduction or trials categorised as ‘gene therapy’.

mRNA is a promising alternative to pDNA. As shown in Fig. 5, the very early onset of protein expression from mRNA provides a significant advantage over pDNA because mRNA does not need to be delivered into the nucleus. It is still challenging to obtain prolonged protein expression from mRNA to a comparable level as pDNA. Nevertheless, mRNA has simpler intracellular processes compared with pDNA. Thus, once the instability of mRNA can be sufficiently overcome by the polyplex nanomicelle, mRNA is likely to be advantageous to satisfy the various demands of therapeutic applications in a more flexible manner.

In this context, it is essential to regulate the immunogenicity of mRNA. The results of analysing immune responses both *in vivo* and *in vitro* (Fig. 3, 4) strongly suggested that the polyplex nanomicelle effectively suppressed the immune responses even when a considerable amount of mRNA was introduced into the cells. According to the results of naked mRNA, mRNA induced significant responses even with a very low amount of cellular uptake. The modification of mRNA could indeed reduce immune responses; however it could not eliminate them completely. Eventually, the nanomicelle played an effective role in reducing immune responses even when unmodified mRNA was used. This result should be attributed to the stealth property of the nanomicelle of encapsulating mRNA shielded by the outer PEG layer [27]. Furthermore, the cationic polymer used in the polyplex nanomicelle PAsp(DET) has a strong capacity to promote the endosomal escape [32], [40], thereby allowing the polyplex containing mRNA to smoothly travel through the endosomes without being recognised by TLRs.

Of noted, Lipofectamine 2000 induced strong immune responses after transfection toward 293-hTLR9 as well as 293-hTLR7 cells, although responses in the former were lower than those in the latter (Fig. 4). It is known that lipid-based reagents tend to destabilise the plasma membrane, facilitating the smooth internalisation of lipoplexes [41]. In addition, the facile disintegration of the lipoplexes to release the mRNA inside the cells contributed to efficient protein expression in *in vitro* settings [42]. However, these properties of lipoplexes may also increase the mRNA recognition by TLRs inside the cells. Furthermore, it is likely that not only specific mRNA recognition by TLR7 but a different factor that could affect TLR9, presumably the leakage of genomic DNA from other cells because of membrane destabilisation, were involved in immune responses observed after transfection using Lipofectamine 2000.

As demonstrated in this study, the polyplex nanomicelle successfully provided protein secretion into CSF continuously for up to 3 days after administration. To the best of our knowledge, this is the first report of prolonged protein expression in CNS for more than a few days by *in vivo* mRNA administration using a non-viral delivery system. The key feature responsible for these outcomes should be attributed to the almost complete suppression of mRNA immunogenicity, regardless of the modification of mRNA. The stable retention of mRNA inside the nanomicelle and smooth endosomal escape may contribute to reduce unfavorable recognition of mRNA by TLRs in the endosomes. In the future, the retention and release kinetics of mRNA inside the nanomicelle should be highly controlled by the sophisticated molecular design of the nanomicelle, as was the case in our previous efforts for the nanomicelle containing pDNA or short interfering RNA (siRNA) [40]. In this manner, we believe that even more prolonged expression from mRNA will be achieved using the polyplex nanomicelle system to fulfill the various needs of treatments and administration routes (systemic or local injection), opening the door to various new therapeutic strategies using mRNA.

## Materials and Methods

### Preparation of mRNA

For *in vitro* transcription (IVT) of luciferase, Gluc and GFP, the protein-expressing fragment of pGL4.13 (Promega, Madison, WI, USA), pCMV-Gluc control plasmid (New England BioLabs, Ipswich, MA, USA), and AcGFP vector (Clontech, Mountain View, CA, USA) respectively, were cloned into pSP73 vector (Promega) to give expression under a T7 promoter. pDNA was used as template for IVT after linearization by Nde I. IVT was performed using the mMESSAGE mMACHINE T7 Ultra Kit (Ambion, Invitrogen, Carlsbad, CA, USA), followed by polyadenylation using the poly(A) tail kit (Ambion). For mRNA modification, 5-methyl-CTP, pseudo-UTP and 2-thio-UTP (TriLink BioTechnologies, San Diego, CA, USA) were added to the reaction solution at compositions of 20%, 10% and 10% in total CTP or UTP, respectively, following the procedure reported previously [10]. Transcribed mRNA was purified with the RNeasy Mini Preparation Kit (Qiagen, Hilden, Germany). The mRNA concentration was determined spectroscopically at 260 nm.

### Constructs for pDNA delivery

A protein-expressing fragment of pCMV-Gluc control plasmid were cloned into pCAG-GS (RIKEN, Tokyo, Japan) to provide expression under a CAG promoter/enhancer.

### Preparation of polyplex nanomicelle and other carriers containing mRNA or pDNA

The PEG–PAsp(DET) block copolymer and PAsp(DET) homo polymer were synthesized as reported previously [30]. The PEG used in this study had a molecular weight (MW) of 12,000. By ^1^H-NMR analyses, the polymerization degree of the PAsp(DET) portion was determined to be 57 for PEG–PAsp(DET) and 52 for PAsp(DET). Linear polyethyleneimine (LPEI) (ExGen 500 *in vivo*; MW = 22 kDa) was purchased from MBI Fermentas (Burlington, ON, Canada). For the preparation of the polyplex nanomicelle, PEG-PAsp(DET) polymer and nucleic acids (mRNA or DNA) was separately dissolved in 10 mM Hepes buffer. At this stage, the concentration of nucleic acid was set to 300 µg/ml, and that of PEG-PAsp(DET) was adjusted to obtain the ratio of amino groups in polymers to phosphate in mRNA or DNA (N/P ratio) to be 8. The solutions of PEG-PAsp(DET) polymer and nucleic acids were mixed by the volume ratio of 1∶2, resulting in the polyplex nanomicelle solution containing 200 µg/ml of nucleic acids. PAsp(DET)-based mRNA carrier (N/P = 8) was prepared similarly as the polyplex nanomicelle. LPEI-based mRNA carrier was prepared following the manufacture's protocol at N/P ratio of 6. Lipofectamine 2000 (Invitrogen) and mRNA were mixed at the ratio indicated in the manufacturer's protocol. Final concentrations of nucleic acids (mRNA and pDNA) were adjusted to 200 µg/ml for all the samples.

### Characterization of polyplex nanomicelle

The size and polydispersity index (PDI) of polyplex nanomicelle was measured by the dynamic light scattering measurement using Zetasizer Nanoseries (Malvern Instruments Ltd., UK) at a detection angle of 173° and a temperature of 25°C. After 3 times measurement of the sample, the data derived from the rate of decay in the photon correlation function were treated by a cumulant method, and the corresponding diameter of each sample was calculated according to the Stokes-Einstein equation. The nanomicelle was determined to have the size of 50.0 nm with a narrow distribution of polydispersity index = 0.19 (Fig. S1)

### Preparation of Gluc protein solution

For the preparation of Gluc protein solution, *in vitro* transfection of GLuc-expressing pDNA was performed and the secreted GLuc protein was collected. HuH-7 cells were seeded at a density of 160,000 cells/well in 6-well culture plates. After 24 h of incubation in Dulbecco's Modified Eagle Medium (DMEM) (Sigma–Aldrich, St. Louis, MO, USA) containing 10% fetal bovine serum (FBS) (Life Technologies Japan Ltd., Tokyo, Japan) and 1% penicillin/streptomycin (Sigma–Aldrich), PAsp(DET)-based carriers loading pCAG-Gluc pDNA (N/P = 10) were added to each well (8 µg/well). After 24 h of transfection, the culture medium was replaced with phosphate-buffered saline (PBS), followed by incubation for 6 h. Following this, PBS containing GLuc protein was recovered and used as Gluc protein solution.

### Intrathecal injection of carrier solution containing mRNA

BALB/c mice (female, 7 weeks old) and SD(IGS) rats (female, 8 weeks old) were purchased from Charles River Laboratories (Yokohama, Japan). Administration to neural tissues of mice was performed as described previously [43]. In brief, the mice were anaesthetized with 3% isoflurane (Abbott Japan Co., Ltd., Tokyo, Japan) and placed in a prone position with the neck bent forward. A 30-gauge needle was inserted into the cisterna magna from the space between occiput and C1, and 10 µl of solution containing 2 µg of mRNA was injected in 60 sec. For rats, meninges between occiput and C1 were exposed after anesthetizing by 3% isoflurane. A 30-gauge needle was inserted into the cisterna magna, and 50 µl of nanomicelle solution containing 10 µg of mRNA or pDNA, or Gluc protein solution was injected in 60 sec. CSF was collected from the cisterna magna in a similar manner by inserting a needle between occiput and C1. All animal protocols were conducted with the approval of the Animal Care and Use Committee, University of Tokyo, Japan.

### Evaluation of luciferase expression in neural tissues

For evaluation of luciferase in the mice, the brain and spinal tissues were excised and thoroughly homogenized using a Multi-beads shocker (Yasui Kikai Corporation, Osaka, Japan). Luciferase expression was measured by the Luciferase assay system (Promega) using the Lumat LB9507 luminometer (Berthold, Bad Wildbad, Germany). The expression was represented after normalisation by total protein concentrations in the tissue lysates. For evaluation of GLuc (a secreted type of luciferase) in rats, CSF was collected, followed by measurement of expression using the Renilla Luciferase Assay system (Promega) and the Lumat LB9507 luminometer. Relative luminescence unit (RLU) value at 0 h of protein administration was plotted as 1/7 of RLU of the injected solution (50 µl) prepared from culture cells (described previously) because CSF volume of an adult rat is approximately 300 µl [44] and the solution was assumed to be diluted by 7 times. Indeed, it was confirmed that the CSF sample collected just after the administration of GLuc protein showed the expected RLU value of 1/7 of the injected solution (n = 1).

### Immunohistological evaluation of neural tissue to analyse GFP expression

Brain tissues were harvested at 48 h after GFP-expressing mRNA administration, and 5-µm-thick frozen section were prepared by a method using an adhesive film [45]. GFP was immunostained with an anti-GFP monoclonal antibody (Invitrogen) at a dilution of 1∶500 and an Alexa488-conjugated secondary antibody (Invitrogen, Carlsbad, CA, USA). After staining the nuclei with Hoechst 33342 (Dojindo, Kumamoto, Japan), the sections were observed with an Axiovert 200 fluorescence microscope (Carl Zeiss, Jena, Germany) using a 20× EC Plan Neofuar objective (Carl Zeiss).

### Evaluation of immune responses in neural tissues

Total RNA was isolated from extracted neural tissues using the RNeasy Mini Preparation Kit. Gene expression of cytokines and interferons was analysed by RT-PCR using an ABI Prism 7500 Sequence Detector (Applied Biosystems, Foster City, CA, USA), and TaqMan Gene Expression Assays (Applied Biosystems, Mm00446190_m1 for interleukin (IL)-6, Mm00443258 for tumour necrosis factor (TNF)-α, Mm00439552_s1 for interferon (INF)-β1, Mm00833969_s1 for IFN-α4 and Mm00607939 for β-actin).

### Analyses of exogenous mRNA recognition by TLRs in 293-hTLR7 and 293-hTLR9 cells

HEK293 cells stably transformed to express human TLR7 and TLR9 (InvivoGen, San Diego, CA, USA) were seeded at a density of 400,000 cells/well in 6-well culture plates and incubated in DMEM containing 10% FBS and 1% penicillin/streptomycin. After 24 h of incubation, the medium was replaced with serum free Opti-MEM medium (Invitrogen), and solution containing 8 µg of RNA encoding luciferase was added to each well. At 4 h after the addition of mRNA, total RNA was isolated from the cells using the RNeasy Mini Preparation Kit. Gene expression was analysed by RT-PCR using TaqMan Gene Expression Assays (Hs00174103_m1 for IL-8, Hs01077958_s1 for INF-β1 and 4310881E for β-actin). The cellular uptake of transfected mRNA was also quantified with RT-PCR by amplifying a 117-bp sequence in the luciferase gene using a forward primer, TGCAAAAGATCCTCAACGTG, and reverse primer, AATGGGAAGTCACGAAGGTG.

## Supporting Information

Figure S1 **Size distribution of polyplex nanomicelle determined by dynamic light scattering (DLS).**
(PDF)

Figure S2 **Tissue distribution of luciferase expression after polyplex nanomicelle administration.** Luciferase was extracted from the central nervous system (CNS) of mice at 4 h (closed bar) and 24 h (open bar) after the administration. The data are presented as the mean ± standard error of the mean (s.e.m.) (N = 6). RLU, relative luminescence units.
(PDF)

Figure S3 **Cellular uptake of messenger RNA (mRNA) after** ***in vitro*** **delivery.** HEK293 cells expressing Toll-like receptor (TLR) 7 were treated with naked mRNA, polyplex nanomicelle and Lipofectamine 2000-based carrier from unmodified mRNA. The amount of mRNA uptake was quantified at 4 h after the treatment using real-time quantitative PCR (RT-PCR). The data are presented as the mean ± standard error of the mean (s.e.m.) (N = 6).
(PDF)
