# Supplementary material for: In Vivo Messenger RNA Introduction into the Central Nervous System Using Polyplex Nanomicelle
Source: PLoS One. 2013 Feb 13;8(2):e56220. doi: 10.1371/journal.pone.0056220 (PMC3571986; doi:10.1371/journal.pone.0056220)
Supplement: Figure S1 — Size distribution of polyplex nanomicelle determined by dynamic light scattering (DLS). (PDF) [file pone.0056220.s001.pdf]

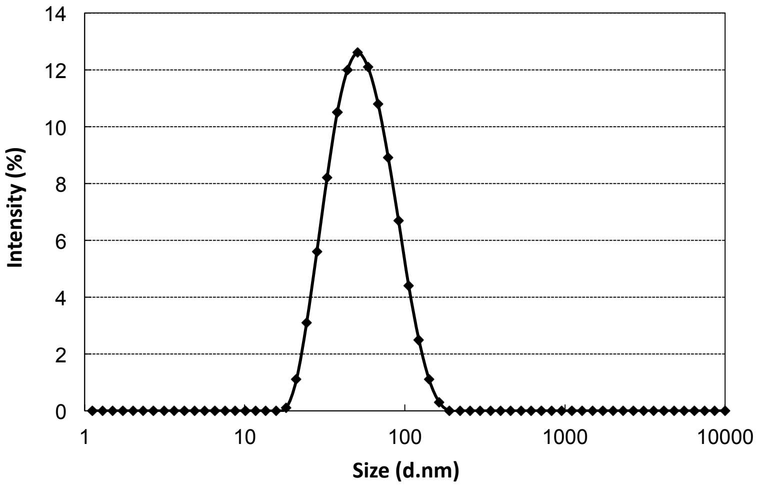

Figure S1 Size distribution of polyplex nanomicelle determined by dynamic light scattering (DLS).
