# Supplementary material for: In Vivo Messenger RNA Introduction into the Central Nervous System Using Polyplex Nanomicelle
Source: PLoS One. 2013 Feb 13;8(2):e56220. doi: 10.1371/journal.pone.0056220 (PMC3571986; doi:10.1371/journal.pone.0056220)
Supplement: Figure S2 — Tissue distribution of luciferase expression after polyplex nanomicelle administration. Luciferase was extracted from the central nervous system (CNS) of mice at 4 h (closed bar) and 24 h (open bar) after the administration. The data are presented as the mean ± standard error of the mean (s.e.m.) (N = 6). RLU, relative luminescence units. (PDF) [file pone.0056220.s002.pdf]

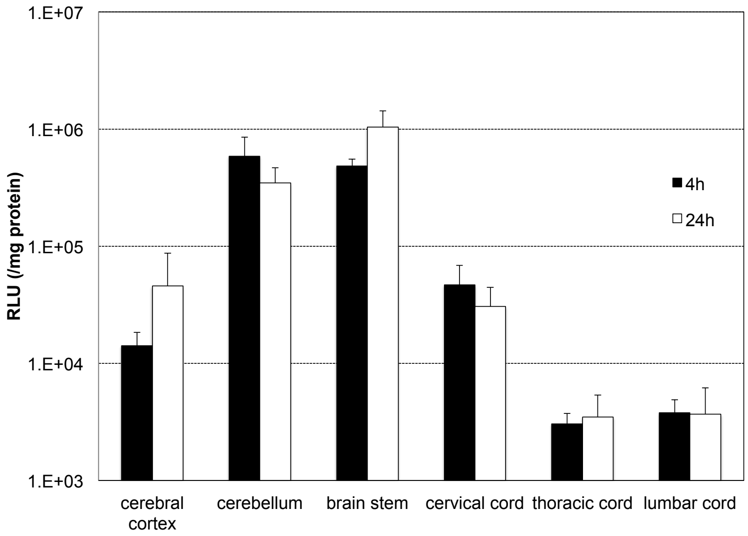

Figure S2 Tissue distribution of luciferase expression after polyplex nanomicelle administration.

Luciferase was extracted from the central nervous system (CNS) of mice at 4 h (closed bar) and 24 h (open bar) after the administration. The data are presented as the mean ± standard error of the mean (s.e.m.) (N = 6). RLU, relative luminescence units.
