# Supplementary material for: In Vivo Messenger RNA Introduction into the Central Nervous System Using Polyplex Nanomicelle
Source: PLoS One. 2013 Feb 13;8(2):e56220. doi: 10.1371/journal.pone.0056220 (PMC3571986; doi:10.1371/journal.pone.0056220)
Supplement: Figure S3 — Cellular uptake of messenger RNA (mRNA) after in vitro delivery. HEK293 cells expressing Toll-like receptor (TLR) 7 were treated with naked mRNA, polyplex nanomicelle and Lipofectamine 2000-based carrier from unmodified mRNA. The amount of mRNA uptake was quantified at 4 h after the treatment using real-time quantitative PCR (RT-PCR). The data are presented as the mean ± standard error of the mean (s.e.m.) (N = 6). (PDF) [file pone.0056220.s003.pdf]

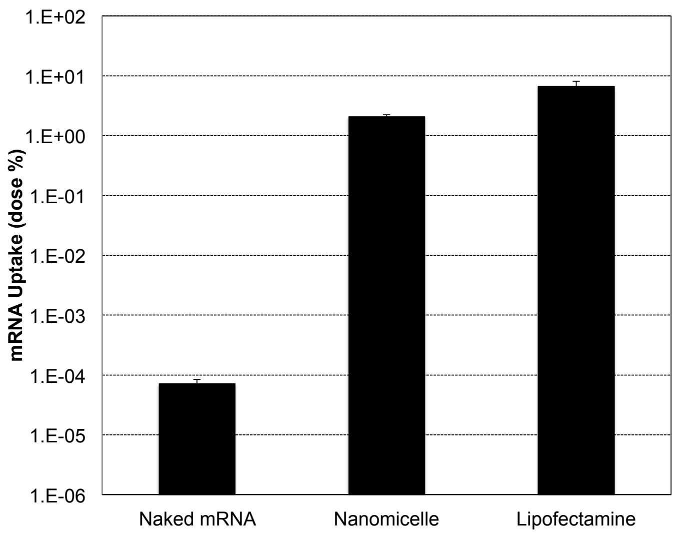

Figure S3 Cellular uptake of messenger RNA (mRNA) after *in vitro* delivery.

HEK293 cells expressing Toll-like receptor (TLR)7 were treated with naked mRNA, polyplex nanomicelle and lipofectamine 2000-based carrier from unmodified mRNA. The amount of mRNA uptake was quantified at 4 h after the treatment using real-time quantitative PCR (RT-PCR). The data are presented as the mean ± standard error of the mean (s.e.m.) (N = 6).
